# Bilirubin ameliorates murine atherosclerosis through inhibiting cholesterol synthesis and reshaping the immune system

**DOI:** 10.1186/s12967-021-03207-4

**Published:** 2022-01-03

**Authors:** Guanmei Wen, Leyi Yao, Yali Hao, Jinheng Wang, Jinbao Liu

**Affiliations:** 1 Guangzhou Institute of Cardiovascular Disease, Guangdong Key Laboratory of Vascular Diseases, State Key Laboratory of Respiratory Disease, The Second Affiliated Hospital, Guangzhou Medical University, Guangzhou, 510260 Guangdong China; 2 Guangzhou Municipal and Guangdong Provincial Key Laboratory of Protein Modification and Degradation, School of Basic Medical Sciences, Affiliated Cancer Hospital & Institute of Guangzhou Medical University, Guangzhou Medical University, Guangzhou, 511436 China; 3 Institute of Digestive Disease of Guangzhou Medical University, The Sixth Affiliated Hospital of Guangzhou Medical University, Qingyuan People’s Hospital, Qingyuan, 511500 Guangdong China

**Keywords:** Bilirubin, Atherosclerosis, 3-Hydroxy-3-Methylglutaryl-CoA Reductase, Myeloid-derived suppressor cells, Natural killer cells

## Abstract

Atherosclerosis is a chronic inflammatory disease caused mainly by lipid accumulation and excessive inflammatory immune response. Although the lipid-lowering and cardioprotective properties of bilirubin, as well as the negative relationship between bilirubin and atherosclerosis, were well documented, it is not yet clear whether bilirubin can attenuate atherosclerosis in vivo. In this study, we investigated the role of bilirubin in improving atherosclerosis. We found that mildly elevated bilirubin significantly reduced the risk factors of atherosclerosis, such as plasma glucose, total cholesterol, and low-density lipoprotein cholesterol, and the formation of atherosclerotic plaques, liver total cholesterol, and cholesterol ester concentration in apolipoprotein E-deficient (ApoE^−/−^) mice fed a western-type (high fat) diet. It was further found that bilirubin could promote the degradation of 3-Hydroxy-3-Methylglutaryl-CoA Reductase (HMGCR), a rate-limiting enzyme for endogenous cholesterol synthesis. Using mass cytometry-based high dimensional single cell analysis, we observed a decrease of natural killer cells and an increase of dendritic cells and myeloid-derived suppressor cells, which all are closely associated with atherosclerosis risk factors and contribute to the improvement of atherosclerosis, in ApoE^−/−^ mice treated with bilirubin. By in-depth analysis, modulation of multiple spleen or peripheral blood T cell clusters exhibiting either positive or negative correlations with total cholesterol or low-density lipoprotein cholesterol was detected after bilirubin treatment. In this study, we demonstrate that bilirubin serves as a negative regulator of atherosclerosis and reduces atherosclerosis by inhibiting cholesterol synthesis and modulating the immune system.

**Supplementary Information:**

The online version contains supplementary material available at 10.1186/s12967-021-03207-4.

## Introduction

Cardiovascular disease (CVD) has become the leading cause of death and morbidity worldwide [1]. Atherosclerosis is the principal cause of CVDs, cerebral infarctions, and peripheral vascular diseases. Atherosclerosis is defined as a chronic inflammatory disease of the arteries. Plaque decomposition or plaque rupture can block arteries, causing severe acute cardiovascular events such as myocardial infarction, stroke, and sudden death [2–5].

Bilirubin is a final product of heme catabolism. In microsomes, heme is converted into carbon dioxide, iron, and biliverdin IX (biliverdin) and thereafter biliverdin reductase transforms biliverdin into bilirubin [6]. The physiological concentration of bilirubin is beneficial for oxidative stress-related pathology, a key event in the development of atherosclerosis [7–9]. Bilirubin levels have been shown to be inversely correlated with CVD mortality and severity [10–16]. Numerous studies have shown that bilirubin offers cardioprotective properties in Gilbert syndrome (GS) patients, who suffer sustained unconjugated hyperbilirubinemia. A low prevalence (2%) of ischemic heart disease (IHD) was found in GS patients, as compared to a higher prevalence (12.1%) among the general population [17]. Hyperbilirubinemia in GS subjects or Gunn rats causes a decrease in circulating cholesterol, triacylglycerol, and oxidized low-density lipoprotein (LDL) by increasing the serum anti-oxidative functions [18, 19]. Bilirubin has the potential to lower lipid, which is associated with cardiovascular protection in GS, as well as dyslipidemia, one of the leading risk factors for atherosclerosis [20]. Oxidative stress plays a critical role in the pathogenesis of atherosclerosis. Bilirubin, a natural endogenous antioxidant, protects vascular cells from oxygen radical damage, inhibits oxidative modification of LDL and improves lipoprotein composition [21, 22]. Bilirubin also inhibits inflammation by downregulating the expression of endothelial cell adhesion molecules, preventing leukocyte adhesion, rolling, and infiltration into vessels [23–25]. Additionally, bilirubin reduces neoplastic intima formation by inhibiting smooth muscle cell proliferation and migration. Because of these properties, bilirubin is involved in every aspects of atherosclerosis, inhibiting plaque formation and promoting plaque stabilization [26, 27]. These findings greatly demonstrate the importance of bilirubin in the regulation of lipids and improvement of atherosclerosis. To date, the underlying mechanisms of hypocholesterolemia in hyperbilirubinemia have not been fully elucidated.

Atherosclerosis is a disease associated with inflammation that relies heavily on the immune system for its development and modulation [28]. New high-throughput single cell technologies, such as mass cytometry and single-cell RNA sequencing, enable a comprehensive analysis of the immune system during atherosclerosis progression through uncovering the diversity of > 15 immune cell populations [29–31]. The dissection of immune modulation has greatly contributed to the understanding of atherosclerosis mechanisms and has provided novel insights into its treatment via immune therapy. The hypolipidemic effect of bilirubin led us to examine its effect on the development of atherosclerosis in an apolipoprotein E-deficient (ApoE^−/−^) mouse model, as well as the mechanisms involved from the perspective of lipid regulation and immunomodulation using mass cytometry-based single cell analysis. In the present study, we found that bilirubin negatively regulates atherosclerosis through inhibiting cholesterol synthesis and modulating the immune system.

## Methods

### Experimental animals

Animal experiments in our study were approved by the Institutional Committee for the Use and Care of Laboratory Animals of Guangzhou Medical University (2017-014) and performed in accordance with the guidelines from Directive 2010/63/EU of the European Parliament on the protection of animals used for scientific purposes or the NIH guidelines. 8-week-old male ApoE^−/−^ mice (C57BL/6.129P2-APOE/J, Beijing Vital River Laboratory Animal Technology Co., Ltd) were divided into two subgroups. One group (Bilirubin, n = 14) received an intraperitoneal injection of bilirubin (20 mg/kg/d, B4126, Sigma) every other day for 10 weeks. Mice injected with the vehicle served as a control (Control, n = 8). Both groups started the western-type diet supplemented with 21% fat (wt/wt) and 0.15% cholesterol (wt/wt), simultaneously receiving the injection of bilirubin or vehicle. All mice were maintained on these diets for 10 weeks. The body weight, diet, and drinking of mice were monitored for a continuous week. The mice were euthanized with an overdose of pentobarbital sodium (200 mg/Kg) for tissue collection.

### Biochemical assays

Peripheral blood was collected into a heparin-coated tube by cardiac puncture and centrifuged to isolate plasma. Total bilirubin (T-BIL) in the plasma was determined by the vanadic acid oxidation method. Liver enzymes, including alanine aminotransferase (ALT) and aspartate aminotransferase (AST), were measured by the ultra-violet lactate and malate dehydrogenase methods. Total cholesterol and triacylglycerol levels were measured by an oxidase method. High-density cholesterol (HDL-C) and low-density cholesterol were measured by a direct method. The total bilirubin assay kit was from FUJIFILM Wako pure Chemical Corporation, Japan. The kits for glucose, liver enzymes, lipids, and lipoproteins were from Kehua Bio-Engineering, China. All the spectrophotometric methods were performed according to the manufacture’s instructions using an autoanalyzer.

### Histological and morphometric analysis

The heart was perfused with phosphate-buffered saline. Whole aorta from the heart, extending 5–10 mm after bifurcation of the iliac, including the brachiocephalic trunk, left common carotid artery, and subclavian arteries, were dissected free of fat tissues and opened longitudinally before being fixed in formalin solution at room temperature (RT) for 24 h. After staining with Oil Red O (ORO), aortae were pinned on a black wax plate and photographed under stereoscopy at standardized magnification and illumination. The ORO positive area and entire aorta tree area were quantified using Image J software. Aorta lesion areas were expressed as a percentage of the whole aorta tree area. The aortic root and brachiocephalic trunk were serially sectioned into 5 μm cryosections. The first section of the aortic sinus was harvested and collected in pairs on glass slides when all three aortic valves became visible in the lumen of the aorta. Neutral lipids deposition in the lesion was visualized by hematoxylin and eosin (H&E) or ORO staining, respectively. The collagen of the lesion was detected by Masson's Trichrome staining. The liver was dissected and weighed, expressed as a ratio of liver weight to body weight. Liver sections were collected and performed H&E or ORO staining for histological and lipid deposition analysis.

### Immunofluorescence staining

The contents of macrophages and smooth muscle cells in aortic sinus lesions were determined by staining with anti–Galectin 3(ab53082) and anti-α-SMA (ab5694) and detecting with Alexa-conjugated antibodies (ab150073 and ab150080). Nuclei were counterstained with DAPI. All antibodies were purchased from Abcam.

### Total cholesterol, cholesterol ester, and free cholesterol assay

Hepatic total cholesterol and free cholesterol concentration were measured using a tissue total cholesterol assay kit (E1015; Applygen Technologies, Inc.) and free cholesterol assay kit (E1016; Applygen Technologies, Inc.) following the manufacturer's instructions. Cholesteryl ester was quantified by subtracting the free cholesterol values from the total cholesterol value.

### Cell culture and treatment

Human normal hepatocyte LO2 (ATCC) was cultured in Dulbecco's Modified Eagle's Medium (DMEM) containing 10% fetal bovine serum (FBS; Gibco), 1 mM L-glutamine, 100 U/ml penicillin, and 100 μg/ml streptomycin. Hepatocellular carcinoma cell line HepG2 (ATCC) was cultured in RPMI-1640 medium (Gibco, Carlsbad, CA, USA) supplemented with 10% FBS, 1 mM L-glutamine, and 100 U/ml penicillin, and 100 μg/ml streptomycin. Both LO2 and HepG2 were maintained at 37 °C in a humidified atmosphere of 5% CO2. For the dose-dependent assay, cells subcultured in the 6-well plate were treated with various concentrations of bilirubin (0, 3, 6 and 12 μM) for 24 h. For the time-dependent studies, cells were treated with 12 μM bilirubin for 0, 6, 12 and 24 h.

### Real-time PCR

Total RNA from cells was extracted with TRIzol reagent (Invitrogen) and reverse-transcribed using PrimeScript™ RT reagent Kit with gDNA Eraser (Takara) according to the manufacturer’s instructions. Real-time PCR was performed on a StemOne™ Real-Tim PCR System (Applied Biosystem, USA) following TB Green™ Premix Ex Taq ™ (Tli RNaseH Plus, Takara) protocol. The primer pairs were used: 18S, forward 5′-CCCAGTAAGTGCGGGTCATAA-3′, reverse 5′- CCGAGGGCCTCACTAAACC-3′; mouse HMGCR forward 5′-TGATTGACCTTTCCAGAGCAAG-3′, reverse 5′- CTAAAATTGCCATTCCACGAGC-3′. Gene expression was calculated based on the −ΔΔCt method. The relative level of mRNA expression was determined using as reference the geometric mean of human/mice 18S (ThermoFisher Scientific).

### Western blot

Cells or tissues were collected and homogenized in ice-cold RIPA buffer. Lysates were maintained constant agitation for 2 h at 4 °C. Gently aspirate the supernatant to a prechilled tube after the centrifugation at 13,200 rpm for 10 min at 4 °C. The protein level of lysates was determined by the BCA protein assay. An aliquot of proteins was separated by SDS-PAGE and then transferred to the immunoblot PVDF membrane. The immunoblots were incubated with primary antibodies at 4 °C overnight, followed by HRP-conjugated secondary antibodies (1:5000) at RT for 1 h. The signals of targeted proteins were visualized by employing the ECL detection system (Amersham). Primary antibodies used for immunoblots were rabbit polyclonal antibodies against HMGCR (ab174830, diluted 1:1000) and mouse monoclonal antibody against GAPDH (ab8245, diluted 1:1000). All antibodies were purchased from Abcam.

### Co-immunoprecipitation (Co-IP)

Cells were collected and treated with IP lysis buffer containing protease inhibitor cocktails at 4 °C for 30 min. Cell lysates were centrifugated at 13,200 rpm for 10 min at 4 °C to remove cell aggregates. 50 μl of protein extract were dissolved in a DTT-containing loading buffer as an input sample. The remaining protein extract was adjusted to a concentration of 2 μg/μl of Co-IP dilution buffer. 500 μl of protein extract were immunoprecipitated with magnetic beads conjugated with indicated antibodies at RT for 1–2 h in a roller. The beads were washed with prechilled Co-IP washing buffer three times and then heated in 2 × loading buffer for 10 min at 70 °C. After centrifugation at 12,000 rpm for 10 min at 4 °C, the beads were collected by inserting the protein samples in a magnetic stack for 10 min at RT. The well-prepared protein samples were analyzed by western blot. The immunoblots were probed with rabbit polyclonal antibody against HMGCR (ab174830, diluted 1:1000), rabbit polyclonal antibody against ubiquitin (ab179434, diluted 1:5000), and mouse monoclonal antibody against GAPDH (ab8245, diluted 1:1000). All antibodies were purchased from Abcam.

### Protein turn-over assay

To evaluate protein half-life, we treated LO2 and HepG2 cells with or without bilirubin (12 μM) in the presence of cycloheximide at a final concentration of 50 µg/ml to block protein synthesis for the indicated time (0, 6, 12, and 24 h). Protein samples were subjected to western blot to analyze the expression of HMGCR.

### Peripheral blood and spleen cell preparation

The peripheral blood was collected from the heart after the injection of pentobarbital sodium. After mice were sacrificed, the spleen was isolated and spleen cells suspension was separated by gently crushing the spleen. Cells from peripheral blood or spleen were fixed with Fix I buffer (Fluidigm) for 10 min at RT. After washing with cold PBS, red blood cell lysis buffer was used to remove erythrocytes from peripheral blood or spleen cells.

### Cell immunostaining

Peripheral blood or spleen cells were barcoded separately with premade combinations of six different palladium isotopes using the Cell-ID 20-Plex Pd Barcoding Kit (Fluidigm) for 30 min at RT. The samples were washed twice with cell staining buffer and then pooled together. The pooled samples were incubated with anti-CD16/32 (FcR III/II, Biolegend, CA, USA) for 10 min at RT to lower nonspecific antibody binding. These cells were washed twice with cell staining buffer and stained with a cocktail of 26 metal isotope-conjugated antibodies (Additional file 1: Table S1) for 30 min at RT. Stained cells were washed and stained with Intercalator-Ir (Fluidigm) at 4 °C overnight. After washing with cell staining buffer and ddH_2_O, cells were resuspended in ultrapure water supplemented with EQ Four Element Calibration Beads (Fluidigm) and analyzed using Helios mass cytometer (Fluidigm).

### Mass cytometry data processing and analysis

The resulting flow cytometry standard (FCS) files generated by mass cytometry were normalized, randomized and debarcoded using the CyTOF software (Fluidigm). The debarcoded files were uploaded to cytobank.cn, an online platform for cytometry-based single cell analysis. The single cell data without debris and doublets were used for high-dimensional analyses, such as viSNE and FlowSOM.

### Statistical analysis

The Shapiro–Wilk test was used to determine whether the data was normally distributed. A parametric test (Student’s t test) was used to determine the statistical significance between 2 groups if the data is normally distributed, otherwise, a nonparametric test (Mann–Whitney test) was used. Correlation analyses were performed using Person’s correlation coefficient. Error bars represent the mean ± standard deviation (SD). p < 0.05 was regarded as statistically significant.

## Results

### Bilirubin lowers the risk of atherogenesis in ApoE^−/−^ mice

**Fig. 1:**
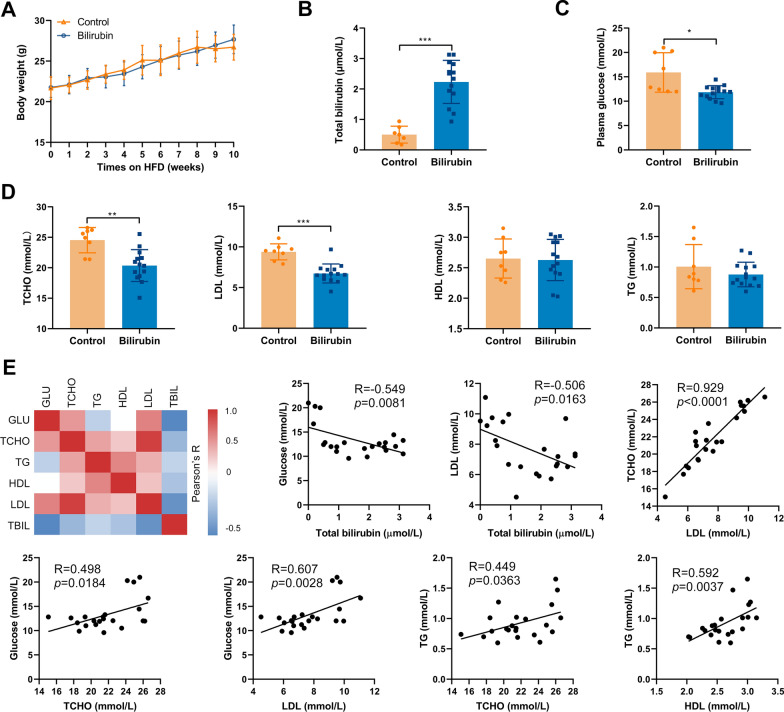
Bilirubin lowers the risk of atherogenesis in ApoE^−/−^ mice. 22 male ApoE^−/−^ mice (8 weeks) fed with a western-type diet were received the intraperitoneal injection of bilirubin (bilirubin group, 20 mg/Kg, n = 14) and vehicle (control group, n = 8) once every other day for ten weeks. **A** The body weight of mice was monitored weekly. **B–D** After 10 weeks of treatment, peripheral blood plasma was collected and the levels of total bilirubin, glucose, total cholesterol (TCHO), low-density lipoprotein cholesterol (LDL), high-density lipoprotein cholesterol (HDL), and total triglyceride (TG) were measured. **E** A heatmap showing the Pearson correlation coefficients for relationships between the concentrations of total bilirubin (TBIL), glucose (GLU), LDL, HDL, TG, and TCHO in peripheral blood. Dot plots (n = 22) showing the Pearson correlation coefficients for relationships between the concentrations of total bilirubin (TBIL), glucose (GLU), LDL, HDL, TG, and TCHO. Error bars represent mean ± standard deviation (SD). Dots represent individual samples, n (control) = 8, n (bilirubin) = 14. *P < 0.05, **P < 0.01 and ***P < 0.001. Differences were determined by an unpaired t-test. Correlations were determined by a Pearson test

To investigate whether bilirubin can protect atherogenesis in vivo, we used a murine model in which ApoE^−/−^ mice were fed on a western-type (high fat) diet. Mice received an intraperitoneal injection of bilirubin or vehicle every other day for 10 weeks. There was no significant difference between the average body weight of the two groups of mice throughout the 10 weeks of intervention. (Fig. 1A). In comparison with a vehicle control group, exogenous bilirubin administered resulted in a fourfold increase in total plasma bilirubin (Fig. 1B). Treatment with bilirubin reduced plasma glucose, total cholesterol, and LDL cholesterol levels in mice. (Fig. 1C and D), whereas both groups had similar levels of triglycerides and high-density lipoprotein (HDL) cholesterol. Furthermore, multiple correlations were found between glucose, total cholesterol, LDL, HDL, triglyceride, and total bilirubin. (Fig. 1E). For example, total bilirubin levels were negatively and significantly correlated with the levels of glucose and LDL levels in the peripheral blood. The LDL level was positively and significantly correlated with the total cholesterol level. Mice with higher levels of glucose also had higher levels of total cholesterol and LDL. There were significant and positive correlations between the concentration of triglyceride and total cholesterol or HDL (Fig. 1E). Considering the long-term exposure, we also investigated the bilirubin toxicity to the liver through histological and biochemical analysis. Bilirubin-treated mice displayed a downward trend in a lower hepatic enzyme activity (AST and ALT) than control mice (Additional file 1: Fig. S1B). But both groups showed no change in liver weight/body weight. (Additional file 1: Fig. S1A). These findings illustrate that a mildly elevated bilirubin reduced the risks of developing atherosclerosis without causing liver toxicity, suggesting a protective role of bilirubin in atherogenesis.

### Bilirubin reduces atherosclerosis in ApoE^−/−^ mice

**Fig. 2:**
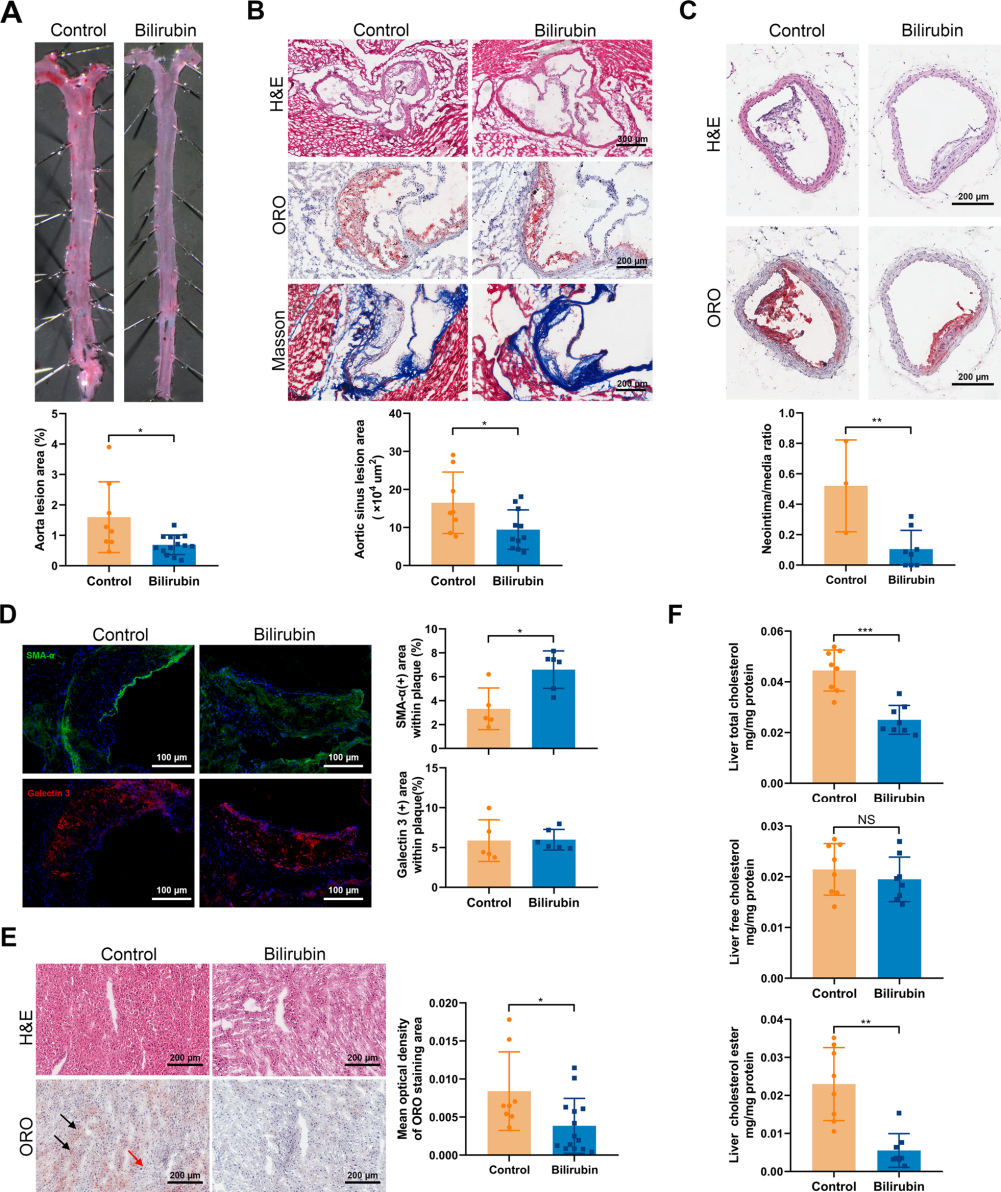
Bilirubin inhibits atherosclerotic lesions formation and hepatic lipid accumulation in ApoE^−/−^ mice. ApoE^−/−^ mice fed with a western-type diet were intraperitoneally injected with (bilirubin, n = 14) or without (control, n = 8) bilirubin. After ten weeks, the animals were sacrificed and the aorta, heart, brachiocephalic artery, and liver tissue were collected for morphometry. **A** Aortas fixed with formalin was performed en face Oil Red O (ORO) staining and the lesion area was quantified. **B** Series of frozen sections of aortic sinus were subjected to hematoxylin–eosin (H&E), ORO, and Masson staining. Aortic sinus lesion area was quantified in the context of H&E staining. **C** Histological analysis of brachiocephalic arteries after H&E and ORO staining. The neointima/media ratio was calculated. **D** Immunofluorescence staining was applied to analyze the distribution of Galectin-3 ( +) (macrophages, red) and α-SMA ( +) (smooth muscle cells, green) in aortic valve lesions. The area of Galectin-3 ( +) and α-SMA ( +) was quantified. **E** Histological analysis of liver after H&E and ORO staining. Area with oil red color are steatosis area. The black arrows indicate the representative steatosis area. The red arrow indicates the normal area. **F** The amount of total cholesterol and free cholesterol in liver lysates was measured. Cholesteryl esters were calculated by subtracting the free cholesterol values from the total cholesterol value. Error bars represent mean ± SD. n (control) = 8, n (bilirubin) = 14. *P < 0.05, **P < 0.01 and ***P < 0.001. Differences were determined by an unpaired t-test

In vivo, bilirubin demonstrated a risk-lowering effect, thus we investigated whether it affects atherosclerotic lesion formation. Morphometric assays were used to measure the aortae and the aortic sinus lesion formation after 10 weeks of intervention. In both groups of mice, lesions were present throughout the aorta, but lesions were reduced in mice treated with bilirubin, as determined by en face ORO staining. (Fig. 2A). The bilirubin treatment significantly decreased the size and lipid load of the aortic sinus plaque and the aortic sinus lesion area. (Fig. 2B). Mice treated with bilirubin also showed reduced brachiocephalic arteries (Fig. 2C). Additionally, bilirubin treatment promoted collagen deposition (Fig. 2B) and α-SMA ( +) smooth muscle cell accumulation without affecting the distribution of Galectin-3( +) macrophages (Fig. 2D). The liver plays a crucial role in lipid metabolism. In the liver, cholesterol is derived from dietary, de novo synthesis, and transport from extrahepatic tissues. In mice fed a western diet for a long time, excessive lipid accumulates in the liver, increasing the risk of atherosclerosis and liver steatosis. H&E and ORO staining showed that bilirubin-treated mice had less hepatic steatosis than vehicle-treated mice (Fig. 2E). Next, The liver was then analyzed using an enzymatic method to determine the content of total cholesterol, cholesterol ester, and free cholesterol. The bilirubin treatment decreased liver total cholesterol and cholesterol ester concentration, but did not affect free cholesterol (Fig. 2F). As a result, bilirubin significantly inhibits the formation of atherosclerotic plaques and favors the phenotype of stable plaque.

### Bilirubin promotes HMGCR protein degradation

**Fig. 3:**
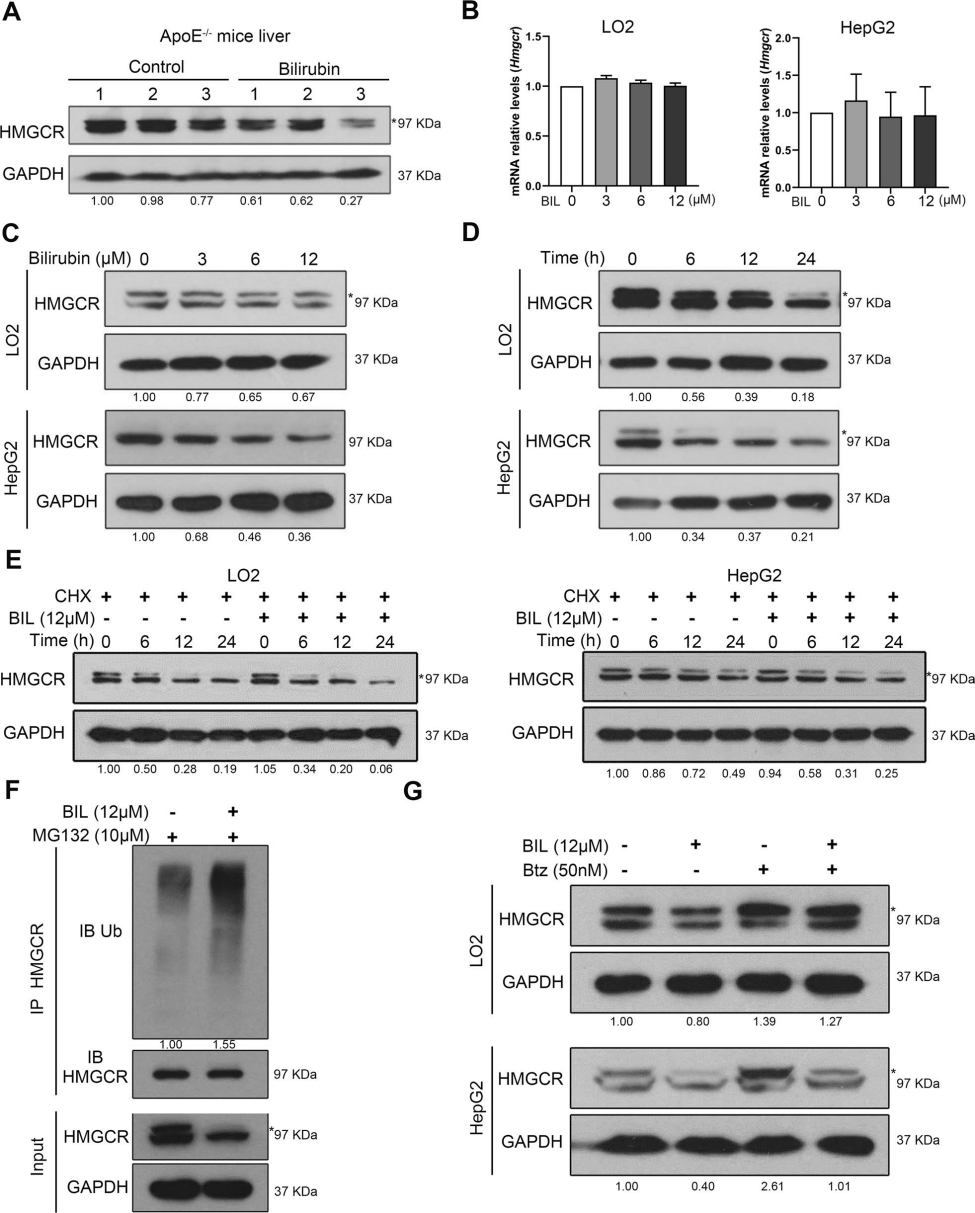
Bilirubin downregulates HMGCR expression in liver and human hepatocytes by promoting the ubiquitination and degradation of HMGCR. **A** Western blot analysis of hepatic HMGCR expression in ApoE^−/−^ mice treated with bilirubin (n = 3) or vehicle (n = 3). **B** Human normal hepatocytes (LO2 or hepatoma cell line (HepG2) were treated with different concentrations of bilirubin (BIL, 0, 3, 6, and 12 μM) for 24 h and the mRNA expression of HMGCR was determined by RT-qPCR. **C** LO2 or HepG2 cells were treated with different concentrations of bilirubin (0, 3, 6, and 12 μM) for 24 h and the protein level of HMGCR was measured by Western blot. **D** LO2 or HepG2 cells were treated with 12 μM of bilirubin for 0, 6, 12, or 24 h and the protein level of HMGCR was measured by Western blot. **E** HepG2 or LO2 cells were incubated with BIL (12 μM) or vehicle in the presence of 50 μg/ml of cycloheximide for the indicated time and the protein level of HMGCR was measured by Western blot. **F** HepG2 cells were treated with or without BIL (12 μM) in the presence of protease inhibitor MG132 (10 μM) for 24 h. Cell lysates were immunoprecipitated with anti-HMGCR antibody and then probed with an antibody against ubiquitin or HMGCR, respectively. **G** LO2 or HepG2 cells were incubated with or without BIL (12 μM) in the presence or absence of protease inhibitor bortezomib (Btz, 50 nM) for 24 h. The expression of HMGCR was determined by Western blot. Representative western blot images of three independent experiments (n = 3) with similar results are shown. Asterisks indicate the correct HMGCR bands. All the bands were quantified and the expression of HMGCR was normalized to GAPDH. Normalized values are below the bands

The liver is the primary organ that synthesizes cholesterol and around 20–25% of total daily cholesterol production occurs here. Since HMGCR is the key rate-limiting enzyme for endogenous cholesterol synthesis, we examined HMGCR expression in vivo and in vitro. HMGCR expression in the liver was significantly reduced in ApoE^−/−^ mice treated with bilirubin compared to vehicle-treated mice (Fig. 3A and Additional file 1: S2A). In some cases, two bands of HMGCR have been detected in our and other studies [32, 33]. The upper one is the HMGCR band and the lower one is the non-specific band. The expression of HMGCR in the liver was also decreased in BALB/c mice treated with bilirubin for three consecutive days (Additional file 1: Fig. S2B). In two hepatic cell lines, bilirubin did not decrease the mRNA expression of HMGCR in vitro (Fig. 3B), but decreased the protein level of HMGCR in a time- and dose-dependent manner (Fig. 3C and D), suggesting that bilirubin may affect HMGCR expression at the translational or post-translational level. After treating two hepatic cell lines with cycloheximide (CHX), we found that bilirubin treatment decreased protein synthesis (Fig. 3E). Considering that HMGCR is degraded through ubiquitination at lysine 89 and 248 [34], we further evaluated the effect of bilirubin on the ubiquitination of HMGCR. Bilirubin increased HMGCR ubiquitination in HepG2 hepatic cells treated with MG132, a proteasome inhibitor (Fig. 3F). Furthermore, bortezomib, a potent protease inhibitor, reversed the decrease in HMGCR levels induced by bilirubin in two hepatic cell lines (Fig. 3G). These results indicate that bilirubin reduces HMGCR level mainly by promoting its ubiquitination and degradation, consequently reducing endogenous cholesterol synthesis.

### Bilirubin affects immune system in the spleen

**Fig. 4:**
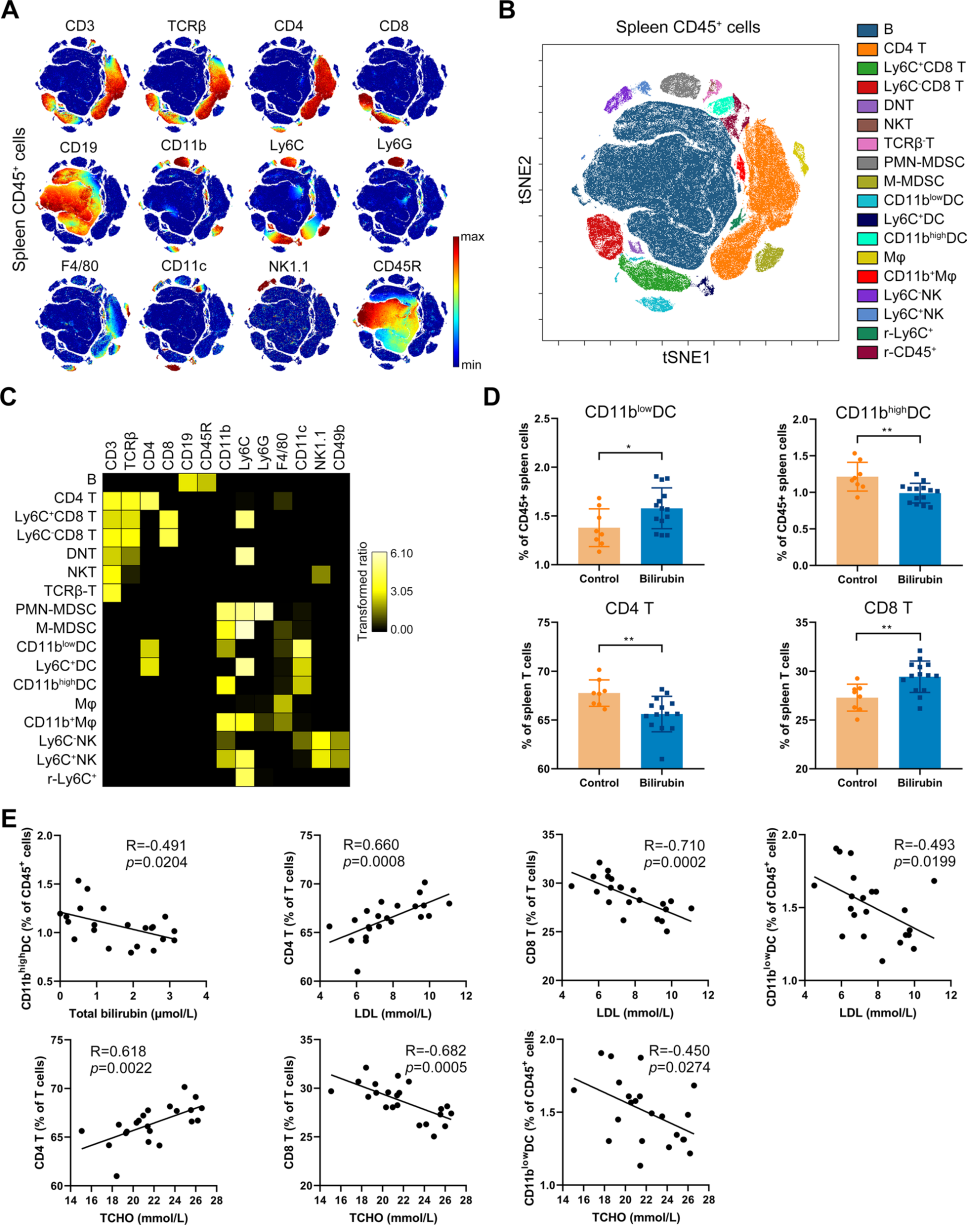
Bilirubin affects immune cells in the spleen. ApoE^−/−^ mice fed with a western-type diet were intraperitoneally injected with (bilirubin, n = 14) or without (control, n = 8) bilirubin. The animals were sacrificed after ten weeks. The spleen cells were collected and simultaneously stained with 26 metal isotope-labeled antibodies. The expression of 26 surface markers was then determined by mass cytometry. **A** viSNE was used to visualize the distribution of the spleen CD45^+^ immune cells from both groups. Cells on the viSNE map were colored by normalized expression of indicated surface markers. **B** 18 cell populations were identified and colored on the viSNE map. **C** A heatmap showing the normalized expression of 13 indicated markers in 18 cell populations. **D** Bar plots showing the frequencies of indicated populations in spleen CD45^+^ or T cells obtained from ApoE^−/−^ mice treated with or without bilirubin. **E** Dot plots (n = 22) showing the Pearson correlation coefficients for relationships between the concentrations of total bilirubin LDL, or TCHO in peripheral blood and the frequencies of indicated cell populations in the spleen. Error bars represent mean ± SD. Dots represent individual samples, n (control) = 8, n (bilirubin) = 14. *P < 0.05, **P < 0.01. Differences were determined by an unpaired t-test. Correlations were determined by a Pearson test

Since bilirubin significantly improves atherosclerosis which is closely associated with the immune response, it is likely that bilirubin also has an effect on the immune system. Thus, we used a high-throughput single cell technology, mass cytometry [31, 35], to simultaneously measure the expression of 26 markers on immune cells from the spleen or peripheral blood at the single cell level. ViSNE analysis of CD45^+^ cells from the spleen was performed to visualize these high-dimensional mass cytometry data in two dimensions. According to the expression of 13 surface markers on the cells displayed on the viSNE map (Fig. 4A), 18 major immune cell populations were identified and gated (Fig. 4B). In each gated population, the expression of 13 markers was the same as the phenotypes of the indicated types of immune cells (Fig. 4C). Next, we compared the proportions of all these cell populations and found that after bilirubin treatment, the percentages of CD11b^low^ dendritic cells (CD11b^low^DCs) significantly increased, while the proportion CD11b^high^DCs markedly decreased (Fig. 4D). In addition, the percentage of CD4 T cells in the spleen T cells was significantly reduced, while the proportion of CD8 T cells increased (Fig. 4D), suggesting that bilirubin induced a change in spleen T cell composition. Interestingly, the proportion of CD4 T cells among spleen T cells was strongly, significantly, and positively related to the LDL and (total cholesterol) TCHO concentration in the blood, while CD8T was strongly, significantly, and negatively related to the LDL and TCHO concentration in blood bilirubin (Fig. 4E), indicating that spleen CD4 T cells are positively associated with atherosclerosis. The proportion of CD11b^high^DC that decreased by bilirubin showed a strong and negatively relationship with total bilirubin (Fig. 4E). Moreover, the proportion of CD8 T cells in spleen T cells, as well as the percentages of CD11b^low^DC, were significantly and negatively associated with the LDL and TCHO concentrations in the blood (Fig. 4E), implicating that these immune cells may favor the improvement of atherosclerosis.

### Bilirubin alters the composition of T cells in the spleen

**Fig. 5:**
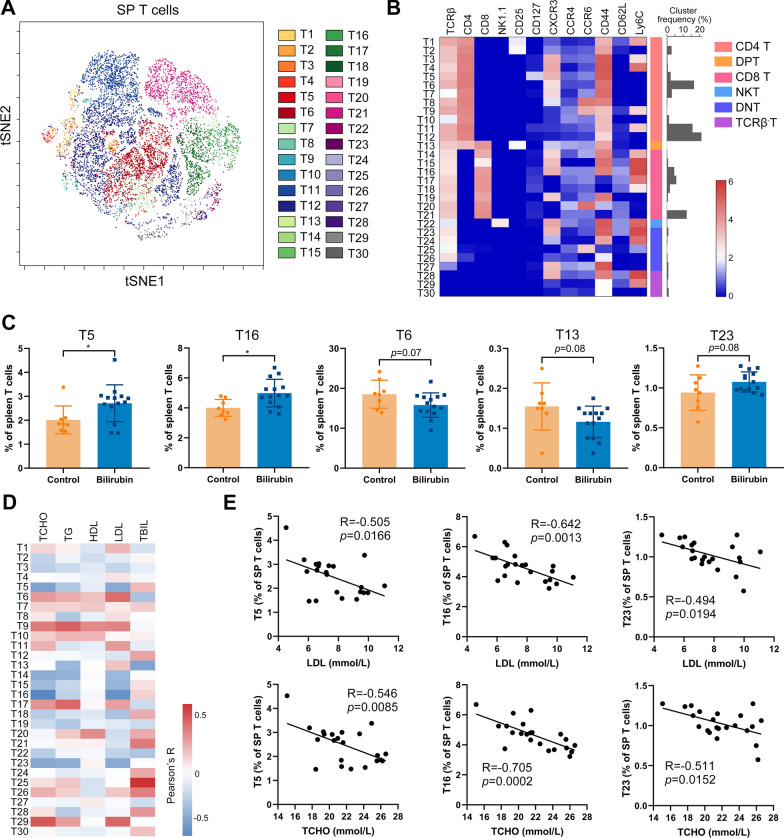
Bilirubin alters the composition of T cells in the spleen. After determining the expression of 26 surface markers on the spleen cells using mass cytometry, the T cells expressing CD3 were gated and analyzed using FlowSOM. 30 T cell clusters were identified using FlowSOM. **A** viSNE was used to visualize the distribution of the spleen T cells. 30 T cell clusters were identified and colored on the viSNE map. **B** A heatmap showing the normalized expression of 12 indicated markers in 30 T cell clusters. **C** Bar plots showing the frequencies of T5, T16, T6, T13, or T23 in spleen T cells obtained from ApoE^−/−^ mice treated with or without bilirubin. **D** A heatmap showing the Pearson correlation coefficients for relationships between the concentrations of total bilirubin (TBIL), LDL, HDL, TG, or TCHO in peripheral blood and the frequencies of indicated spleen T cell clusters. **E** Dot plots (n = 22) showing the Pearson correlation coefficients for relationships between the concentrations of LDL or TCHO in peripheral blood and the frequencies of T5, T16, or T23 clusters. Error bars represent mean ± SD. Dots represent individual samples, n (control) = 8, n (bilirubin) = 14. *P < 0.05. Differences were determined by an unpaired t-test. Correlations were determined by a Pearson test

T cells can influence the process of atherosclerosis by regulating their anti-inflammatory and pro-inflammatory functions and their functions are mainly determined by the direction of their differentiation. T cells are highly heterogeneous and contain various subtypes of differentiated T cells. Thus, we used 12 T cell-related markers to systematically analyze the spleen T cell. After viSNE analysis, heterogeneity in markers was assessed at the single-cell level. From the viSNE map of T cells, we also observed a huge heterogeneity of the T-cell compartments (Additional file 1: Fig. S3A). FlowSOM, an unsupervised cluster analysis for visualizing and interpreting cytometry data, was used on these T cells to map T cell phenotypes exhaustively and to further define any exclusive or significantly altered T cell clusters. 30 T cell clusters containing cells with similar phenotypes were identified and visualized on the viSNE map (Fig. 5A). The expression profiles of all the T cell clusters were visualized in a heatmap (Fig. 5B) and this approach led to the identification of 12 CD4^+^ clusters, one double-positive cluster, eight CD8^+^ clusters, five double-negative phenotypes, one natural killer (NK) T cluster and three TCRβ^−^ clusters (Fig. 5B). Among these 30 T cell clusters, proportions of T5 (CD44^+^CD62L^−^CXCR3^+^CD4T), which mainly contains effector memory (em) Th1 cells, and T16 (Ly6C^+^CD44^+^CD62L^+^CD8T), which mainly contains central memory Ly6C^+^CD8 T (Tcm) cells, were significantly increased after bilirubin treatment (Fig. 5C). In addition, these two clusters, together with T23 which was increased with a trend close to significance, had a mutual, significant, and negative relationship with the concentrations of LDL and TCHO in the blood (Fig. 5D and E). Furthermore, T6 and T13 proportions decreased with a trend close to significance after bilirubin treatment (Fig. 5C). Bilirubin did not affect the proportion of T1 (CD3^+^CD4^+^CD25^+^CD127^−^Ly6C^+^) and T2 (CD3^+^CD4^+^CD25^+^CD127^−^Ly6C^−^) clusters which mainly contain regulatory T (Treg) cells (Additional file 1: Fig. S3B). These data indicate that bilirubin also can regulate the composition of spleen T cells which are related to the improvement of atherosclerosis.

### Bilirubin regulates myeloid-derived suppressor cells (MDSCs), DCs and NK cells in the peripheral blood

**Fig. 6:**
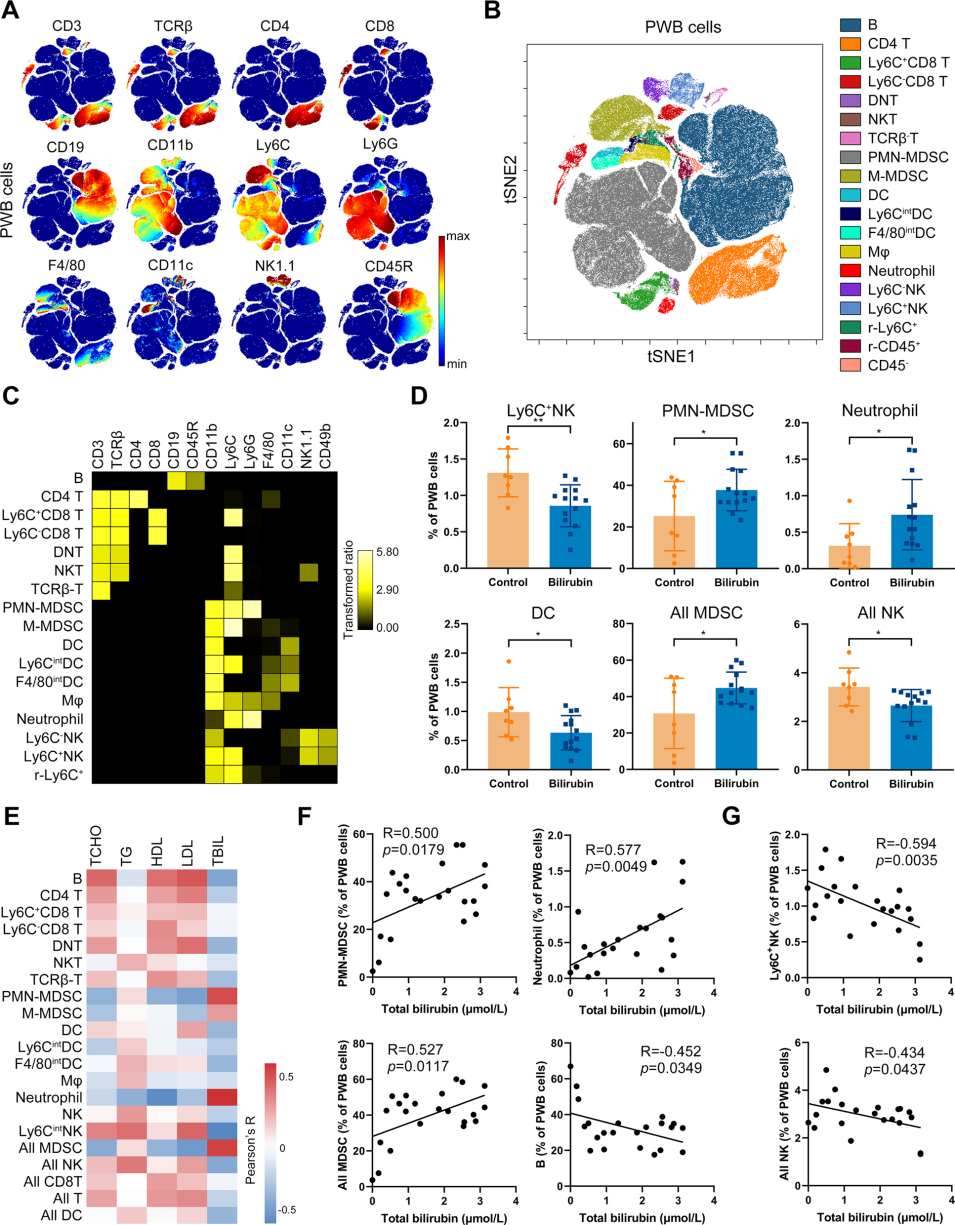
Bilirubin reshapes the immune system in the peripheral blood. ApoE^−/−^ mice fed with a western-type diet were intraperitoneally injected with (bilirubin, n = 14) or without (control, n = 8) bilirubin. The animals were sacrificed after ten weeks. The peripheral white blood (PWB) cells were collected and simultaneously stained with 26 metal isotope-labeled antibodies. The expression of 26 surface markers was then determined by mass cytometry. **A** viSNE was used to visualize the distribution of the PWB cells from both groups. Cells on the viSNE map were colored by normalized expression of indicated surface markers. **B** 19 cell populations were identified and colored on the viSNE map. **C** A heatmap showing the normalized expression of 13 indicated markers in 19 cell populations. **D** Bar plots showing the frequencies of indicated populations in PWB cells obtained from ApoE^−/−^ mice treated with or without bilirubin. **E** A heatmap showing the Pearson correlation coefficients for relationships between the concentrations of TBIL, LDL, HDL, TG, or TCHO in peripheral blood and the frequencies of indicated cell populations. **F** Dot plots (n = 22) showing the Pearson correlation coefficients for relationships between the concentrations of total bilirubin in peripheral blood and the frequencies of indicated cell populations in the PWB cells. **G** Dot plots showing the Pearson correlation coefficients for relationships between the concentrations of total bilirubin in peripheral blood and the frequencies of NK cells. Error bars represent mean ± SD. Dots represent individual samples, n (control) = 8, n (bilirubin) = 14. *P < 0.05, **P < 0.01. Differences were determined by an unpaired t-test. Correlations were determined by a Pearson test

After bilirubin administration, the immune cells in the peripheral blood were also comprehensively analyzed using mass cytometry. viSNE was also used to identify the cell populations (Fig. 6A). 19 immune cell populations were obtained and the expression of their phenotypic markers was confirmed (Fig. 6B and C). The percentages of these 19 cell populations were then compared. Proportions of DC, Ly6C^+^NK, and all NK cells were significantly reduced in the peripheral blood of mice treated with bilirubin (Fig. 6D). The percentages of polymorphonuclear (PMN)-MDSC, neutrophils, and all MDSC significantly increased (Fig. 6D) and were strongly, significantly, and positively correlated with bilirubin concentration (Fig. 6E and F), implicating that bilirubin treatment may directly increase these cells. In addition, the proportion of all MDSC was significantly and negatively correlated with the concentrations of LDL and TCHO in the blood (Additional file 1: Fig. S4A). Considering that higher levels of LDL and TCHO are closely associated with atherogenesis, MDSCs increase by bilirubin may contribute to the improvement of atherogenesis though regulating the levels of LDL and TCHO. Moreover, proportions of Ly6C^+^NK and all NK cells showed a significant negative correlation with blood bilirubin level (Fig. 6G), suggesting that the reduction of these NK cells may be directly caused by bilirubin administration. The proportion of Ly6C^+^NK cells correlated positively and significantly correlated with the level of LDL in the blood (Additional file 1: Fig. S4B). Furthermore, B cells were negatively correlated with the concentration of bilirubin and LDL, and positively associated with TCHO concentration (Fig. 6F and Additional file 1: Fig. S4C). These data propose that bilirubin affects the MDSC and NK cells which are closely associated with the improved atherosclerotic blood lipid profiles.

### Bilirubin mildly affects T cell composition in the peripheral blood

**Fig. 7:**
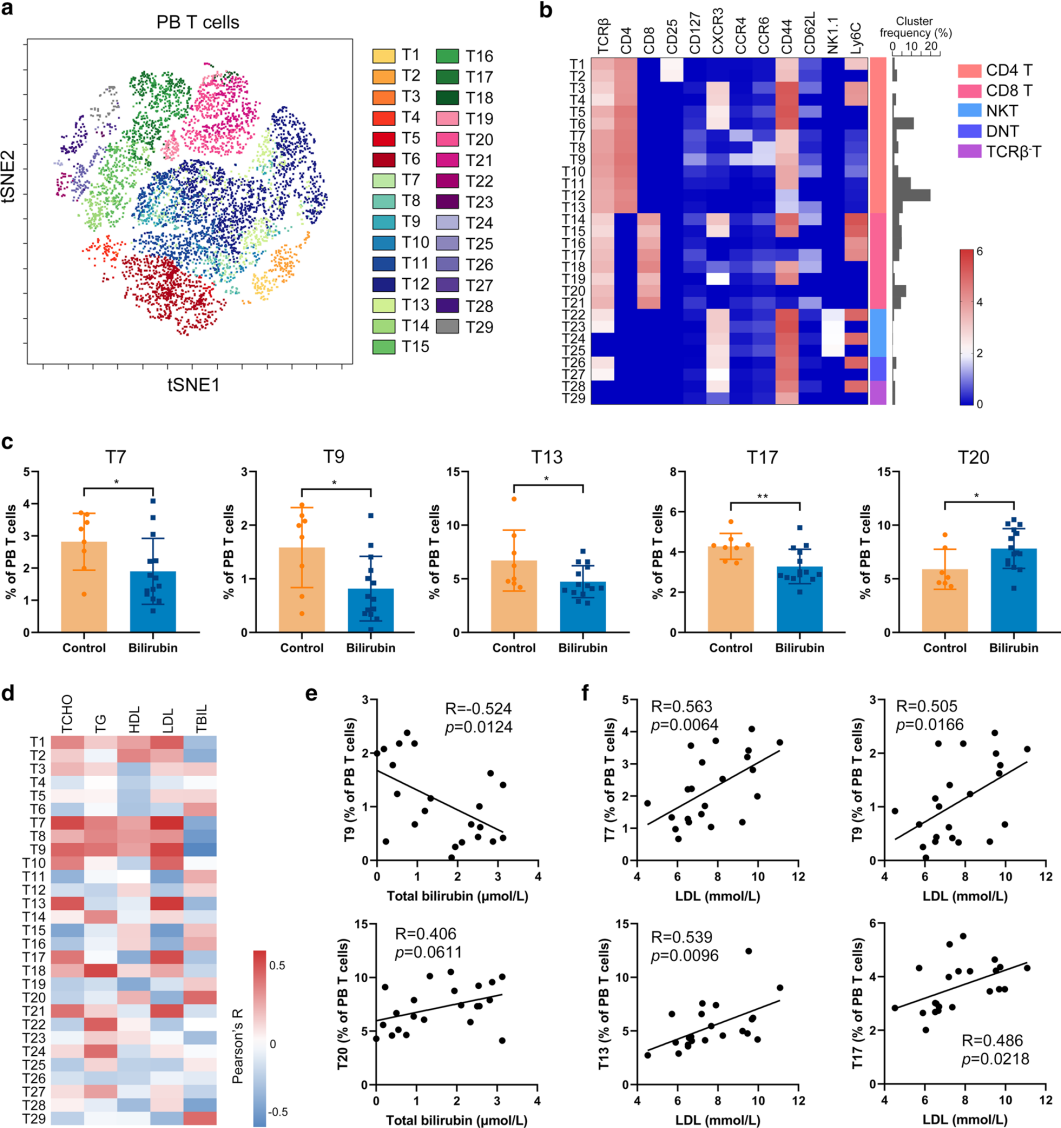
Bilirubin modifies the composition of T cells in the peripheral blood. After determining the expression of 26 surface markers on the PWB cells using mass cytometry, the T cells expressing CD3 were gated and analyzed using FlowSOM. 29 T cell clusters were identified. **A** viSNE was used to visualize the distribution of the peripheral blood T cells. 29 T cell clusters were identified and colored on the viSNE map. **B** A heatmap showing the normalized expression of 12 indicated markers in 29 T cell clusters. **C** Bar plots showing the frequencies of T7, T9, T13, T17, or T20 in peripheral blood T cells obtained from ApoE^−/−^ mice treated with or without bilirubin (left panel). **D** A heatmap showing the Pearson correlation coefficients for relationships between the concentrations of TBIL, LDL, HDL, TG, or TCHO in peripheral blood and the frequencies of peripheral blood T cell clusters. **E** Dot plots (n = 22) showing the Pearson correlation coefficients for relationships between the concentrations of total bilirubin in peripheral blood and the frequencies of T9 or T20 in peripheral blood T cells. **F** Dot plots showing the Pearson correlation coefficients for relationships between the concentrations of LDL in peripheral blood and the frequencies of T7, T9, T13, or T17 in peripheral blood T cells. Error bars represent mean ± SD. Dots represent individual samples, n (control) = 8, n (bilirubin) = 14. *P < 0.05, **P < 0.01. Differences were determined by an unpaired t-test. Correlations were determined by a Pearson test

Peripheral blood T cell composition after bilirubin treatment was also analyzed using viSNE (Additional file 1: Fig. S5A). 29 T cell clusters, including 13 CD4^+^ clusters, eight CD8^+^ clusters, four NKT clusters, two double-negative phenotypes, and two TCRβ^−^ clusters were identified (Fig. 7A and B). Treg cell clusters, T1 and T2, were not changed by bilirubin treatment (Additional file 1: Fig. S5B). T7 (CD44^+^CCR4^+^CD4 T), T9 (CD44^+^CCR4^+^CCR6^+^CD4 T), T13 (CD44^−^CD62L^+^CD4 T), and T17 (Ly6C^+^CD44^−^CD62L^+^CD8 T) were significantly decreased, whereas T20 (Ly6C^−^CD44^−^CD62L^−^CD8 T) was increased after bilirubin treatment (Fig. 7C). Additionally, proportions of two TCRβ^−^ clusters, T28 and T29, were also changed by bilirubin with a trend close to significance (Additional file 1: Fig. S5C). The percentages of T9 and T28 showed a negative and significant correlation with the total bilirubin level, whereas T20 and T29 showed a positive correlation with total bilirubin (Fig. 7D and E and Additional file 1: S5C). There was a positive and significant correlation between the proportions of T7, T9, T13, and T17 and the amount of LDL in the blood. (Fig. 7F). Except for T17, the other clusters were also positively correlated with TCHO concentration (Additional file 1: Fig. S5D). These results suggest that bilirubin can induce changes of T cell subclusters and that these changed T cells also have a strong correlation with the improvement of atherosclerosis.

## Discussion

Accumulating epidemiological evidence has demonstrated the negative relationship between bilirubin and CVD. Bilirubin, a potential predictor and protective marker of CVD, might become a new therapeutic target in the clinical setting. However, little is known about the direct cardioprotective effects of bilirubin. In the present studies, exogenous bilirubin inhibited lesion formation and lowered circulating cholesterol, LDL cholesterol, glucose concentrations, and hepatic lipid accumulation in ApoE^−/−^ mice receiving western-type diet. We confirmed that bilirubin prompted HMGCR degradation via the proteasome pathway, suggesting that the cardioprotective effects of bilirubin may also be governed by its lipid-lowering properties. Furthermore, bilirubin significantly modulated the immune system, which resulted in a cardioprotective immune profile. These findings highlight the negative regulatory role of bilirubin in atherosclerosis while suggesting bilirubin as a potential treatment option for atherosclerosis.

Dyslipidemia and diabetes are common risk factors for atherosclerosis. A higher level of circulating LDL cholesterol or total cholesterol is independently and positively correlated with the prevalence of atherosclerotic peripheral vessel diseases [36]. In our study, there was a significant reduction of total cholesterol (− 17%), LDL-C (− 28%) and blood glucose (− 26%) in mice treated with bilirubin, confirming the lipid-lowering properties of bilirubin. Studies in animals (Gunn rats) or humans (GS subjects) of hyperbilirubinemia have consistently shown that bilirubin protected against IHD and atherosclerosis in dyslipidemia or diabetes high-risk population [17, 19, 37, 38]. In light of this circumstantial evidence, along with our finding showing that bilirubin attenuates atherosclerotic lesion formation and promotes a stable atherosclerotic plaque, further confirm that bilirubin is a negative regulator in atherosclerosis.

Bilirubin protects against atherosclerosis due to its anti-oxidative and anti-inflammatory properties, as well as the regulation of leukocyte migration mediated by endothelial adhesive molecules [7]. In atherosclerosis, foam cells are formed by the uptake of oxidatively modified lipids as oxLDL by macrophages [39]. Bilirubin has been shown to inhibit lipoprotein oxidation and therefore protects endothelial cells from being damaged by oxidative stress [22]. McNamara et al. showed that the serum remnant-like particle cholesterol (RLP-c), an independent risk factor for CVD, was higher in the male patients in the high bilirubin group than those in the low bilirubin group [40]. It is showed that bilirubin regulates cholesterol and lipid metabolism via activating the aryl hydrocarbon receptor (AhR) signaling pathway, which has been shown to protect against cardiogenesis and oxidative stress [41, 42]. Notably, bilirubin, as a novel PPARα agonist, suppressed lipid accumulation in 3T3-L1 adipocytes and PPARα KO mice [43]. According to a recent study, increased unconjugated bilirubin promotes the clearance of excess cholesterol out of the body by promoting transintestinal cholesterol secretion [18]. These previous studies have shown that bilirubin can regulate LDL via multiple pathways, which is also in accordance with our findings.

The liver plays a critical role in maintaining cholesterol homeostasis by regulating multiple mechanisms, including exogenous cholesterol uptake via LDLR and cholesterol biosynthesis via HMGCR activity, esterification for storage, and reverse cholesterol transport (RCT) [44]. A disruption in any one of these pathways can lead to dyslipidemia. Newly synthesized cholesterol assembles into very low-density lipoprotein and triglycerides, which is then excreted into the bloodstream or stored in lipid droplets. Ectopic lipid accumulation and the deposition of lipid within non-adipose tissues (e.g., muscle and liver) contribute to the pathogenesis of metabolic diseases, such as diabetes and atherosclerosis [45, 46]. Hepatic steatosis, an indicator of atherosclerosis, can be caused by excessive lipid deposits in the liver. We found that ApoE^−/−^ mice treated with bilirubin displayed a decrease in lipid droplets accumulation in the liver. In addition, bilirubin also inhibited cholesterol and cholesterol esters production, suggesting that bilirubin attenuates hepatic steatosis and atherosclerosis by interrupting cholesterol biosynthesis. Our findings are consistent with a previous study, which demonstrated mild hyperbilirubinemia conditions significantly reduced the intracellular lipid accumulation in C2C12 skeletal mouse muscle and HepG2 human liver cells [47].

HMGCR, a key rate-limiting enzyme, mediates liver de novo biosynthesis of cholesterol. Statins can significantly suppress cholesterol biosynthesis through inhibiting HMGCR activity and thus are widely used in treating hypercholesterolemia. Two negative feedback pathways are available to regulate the expression of target genes involved in cholesterol metabolism: the processing of sterol regulatory element-binding proteins 2 (SREBP2), a crucial transcriptional regulator of cholesterol biosynthesis, and sterol-induced HMGCR degradation [48]. Here, bilirubin did not reduce the mRNA expression of HMGCR, but significantly decreased its protein level, suggesting the involvement of a post-translational mechanism. Song et al. reported that the mTORC1-USP20-HMGCR axis, a post-transcriptional mechanism, participated in the turnover of HMGCR and that the inhibition of USP20 might be beneficial for a variety of metabolic diseases, such as diabetes, hyperlipidemia, and CVD [48]. Our findings demonstrated that bilirubin promotes the ubiquitination and degradation of HMGCR, thus contributing to a reduction of cholesterol biosynthesis and the improvement of atherosclerosis.

Atherosclerosis is considered a chronic inflammatory disease with autoimmune responses. Both the innate and adaptive immune responses have been implicated in atherosclerosis progression. Here, we found that bilirubin displays atheroprotective properties in ApoE^−/−^ mice and acts as a regulatory role on both innate and adaptive immune cells. DCs present antigens to T cells and thus provide an important bridge between innate and adaptive immunity. DCs involves atherosclerosis through multiple mechanisms, inducing lipid uptake, antigen presentation, and cytokine production [49, 50]. There are three DC subsets, including conventional DC (cDC) 1, cDC2, and plasmacytoid DC [51]. cDC1, which express low levels of CD11b, play atheroprotective function through inducing Treg cells [52]. We showed that bilirubin increases spleen CD11b^low^DCs, which correlates negatively with LDL and TCHO levels. Thus, bilirubin likely exerts atheroprotective effects by regulating the functions of these DCs.

CD4^+^ T cells are important mediators of the adaptive immune response and their subtypes, Th1 cells, have pro-atherogenic functions mainly through secreting pro-inflammatory cytokines, such as IFN-γ, IL-2, IL-3, and tumor necrosis factor [53, 54]. In the present study, CD4 T cells were significantly reduced by bilirubin treatment and this decrease is positively associated with LDL and TCHO, suggesting the involvement of these CD4 T cells in atheroprotective role of bilirubin. Moreover, after in-depth single cell analysis, a decrease of T6, which mainly are Th1 Tem and accounts for above 15% of spleen T cells, contributes to the overall reduction of CD4 T cells. Considering the pro-atherogenic functions of Th1 cells, decrease of effector memory Th1 cell may be one of the causes of bilirubin-induced atherosclerosis improvement.

NK cells, a critical component of the innate immune system, have been demonstrated as an atherogenic factor. NK cells accumulate in the atherosclerotic plaques, and contribute to the expansion of necrotic cores and atherosclerotic lesion development through producing perforin and granzyme B [55]. Antibodies-mediated depletion of NK cells in ApoE^−/−^ mice greatly attenuated atherosclerosis [55]. Another study has also proved the pro-atherogenic role of NK cells by showing that depletion of NK function decreases atherosclerosis in Ldlr^−/−^ mice [56]. Here, bilirubin-induced decrease of NK cells, especially Ly6C^+^NK cells, was observed. Due to the pro-atherogenic functions of NK cells, the reduction of NK cells after bilirubin treatment may benefit the remission of atherosclerosis.

MDSCs comprise immature myeloid cells that consist mainly of progenitors and precursors of granulocytes, macrophages, and dendritic cells [57]. MDSCs primarily mediate immunosuppressive function through inhibiting T cell activation and proliferation, promoting the development of regulatory T cells, blocking antigen recognition, and suppressing NK cell cytotoxicity [57, 58]. Based on MDSC phenotypic and morphological features, two major subsets, named polymorphonuclear (PMN) and monocytic (M)-MDSC, were distinguished. In mice, the expression of CD11b, Ly6G, and Ly6C were introduced to identify these two MDSC subpopulations: PMN-MDSC (CD11b^+^Ly6G^+^Ly6C^low^) and monocytic (M)-MDSC (CD11b^+^Ly6G^−^Ly6C^high^) [59]. MDSCs have been extensively investigated in tumor immunology, and accumulating evidence has demonstrated their involvement in obesity and atherosclerosis [60, 61]. By suppressing T cells, MDSCs are able to reduce atherosclerotic lesion development in Ldlr^−/−^ mice [61]. In our study, bilirubin significantly attenuated the atherosclerosis and caused a significant increase in all MDSC, especially PMN-MDSCs. Moreover, MDSCs are also significantly and negatively correlated with LDL and TCHO, two important markers of atherosclerosis. Thus, bilirubin-induced MDSC increase may also improve atherosclerosis.

Overall, our data clearly show the lipid-lowering and cardioprotective properties of bilirubin in atherosclerosis and that the detailed mechanisms involve inhibition of cholesterol synthesis through promoting HMGCR degradation and regulation of the immune systems. Notably, mass cytometry-based single cell analysis was introduced to comprehensively dissect the regulatory effects of bilirubin on different immune cell lineages and T cell subsets. Further, these comprehensive data suggest that bilirubin negatively regulates atherosclerosis not only by affecting cholesterol synthesis but also by reshaping the immune system. In addition to the methodological novelty of our work, our results directly propose bilirubin as a novel therapeutic strategy for improving atherosclerosis treatment.

## Supplementary Information

**Additional file 1:** **Figure S1.** (A) ApoE^−/−^ mice fed with a western-type diet were intraperitoneally injected with or without bilirubin. After 10 weeks, mice were scarified, and the body and liver weight were measured. The relative liver weight was expressed as liver weight to body weight ratio. (B) Concentrations of liver enzymes were determined by biochemical analysis. Error bars represent mean ± SD. n (control) =8, n (bilirubin) =14. *P < 0.05. Differences were determined by an unpaired t-test. **Figure S2. **(A) Quantification of hepatic HMGCR protein expression in ApoE^−/−^ mice treated with bilirubin (n=3) or vehicle (control, n=3). (B) BALB/c mice were received intraperitoneal injection of bilirubin (20 mg/Kg/day, n=4) or vehicle (n=4) once a day for 3 days and sacrificed at day 4. The expression levels of HMGCR in the livers were determined by western blot. Bar plots showing the relative HMGCR protein expression in the livers (right panel). *P < 0.05. **Figure S3. **After determining the expression of 27 surface markers on the spleen cells using mass cytometry, the T cells expressing CD3 were gated and analyzed using viSNE. viSNE was used to visualize the distribution of the spleen T cells from both groups. Cells on the viSNE map were colored by normalized expression of indicated surface markers. (B) Bar plots showing the frequencies of T1 or T2 in spleen T cells obtained from ApoE^−/−^ mice treated with or without bilirubin. Error bars represent mean ± SD. n (control) =8, n (bilirubin) =14. **Figure S4. **(A) Dot plots showing the Pearson correlation coefficients for relationships between the concentrations of LDL or TCHO in peripheral blood and frequencies of PMN-MDSCs or All MDSCs. (B) Dot plots showing the Pearson correlation coefficients for relationships between the concentrations of LDL in peripheral blood and the frequencies of Ly6C+NK cells. (C) Dot plots (n=22) showing the Pearson correlation coefficients for relationships between the concentrations of LDL or TCHO in peripheral blood and the frequencies of peripheral blood B cells. Correlations were determined by a Pearson test. **Figure S5. **After determining the expression of 27 surface markers on the PWB cells using mass cytometry, the T cells expressing CD3 were gated and analyzed using viSNE. (A) viSNE was used to visualize the distribution of the peripheral blood T cells from both groups. Cells on the viSNE map were colored by normalized expression of indicated surface markers. (B) Bar plots showing the frequencies of T1 or T2 in peripheral blood T cells obtained from ApoE^−/−^ mice treated with or without bilirubin. (C) Bar plots showing the frequencies of T28 or T29 in peripheral blood T cells obtained from ApoE^−/−^ mice treated with or without bilirubin (left panel). Dot plots showing the Pearson correlation coefficients for relationships between the concentrations of total bilirubin in peripheral blood and frequencies of T28 or T29 in peripheral blood T cells. (D) Dot plots (n=22) showing the Pearson correlation coefficients for relationships between the concentrations of TCHO in peripheral blood and the frequencies of T7, T9, T13 in peripheral blood T cells. Error bars represent mean ± SD. n (control) =8, n (bilirubin) =14. Differences were determined by an unpaired t-test. Correlations were determined by a Pearson test. **Table S1.** Mass cytometry antibody reagents.

## Data Availability

All datasets generated for this study are included in the article. The original data that support the findings of this study are available from the corresponding author upon reasonable request.
