# Supplementary material for: Bilirubin ameliorates murine atherosclerosis through inhibiting cholesterol synthesis and reshaping the immune system
Source: J Transl Med. 2022 Jan 3;20:1. doi: 10.1186/s12967-021-03207-4 (PMC8722314; doi:10.1186/s12967-021-03207-4)
Supplement: Supplementary file 1 — Additional file 1: Figure S1. (A) ApoE−/− mice fed with a western-type diet were intraperitoneally injected with or without bilirubin. After 10 weeks, mice were scarified, and the body and liver weight were measured. The relative liver weight was expressed as liver weight to body weight ratio. (B) Concentrations of liver enzymes were determined by biochemical analysis. Error bars represent mean ± SD. n (control) =8, n (bilirubin) =14. *P < 0.05. Differences were determined by an unpaired t-test. Figure S2. (A) Quantification of hepatic HMGCR protein expression in ApoE−/− mice treated with bilirubin (n=3) or vehicle (control, n=3). (B) BALB/c mice were received intraperitoneal injection of bilirubin (20 mg/Kg/day, n=4) or vehicle (n=4) once a day for 3 days and sacrificed at day 4. The expression levels of HMGCR in the livers were determined by western blot. Bar plots showing the relative HMGCR protein expression in the livers (right panel). *P < 0.05. Figure S3. After determining the expression of 27 surface markers on the spleen cells using mass cytometry, the T cells expressing CD3 were gated and analyzed using viSNE. viSNE was used to visualize the distribution of the spleen T cells from both groups. Cells on the viSNE map were colored by normalized expression of indicated surface markers. (B) Bar plots showing the frequencies of T1 or T2 in spleen T cells obtained from ApoE−/− mice treated with or without bilirubin. Error bars represent mean ± SD. n (control) =8, n (bilirubin) =14. Figure S4. (A) Dot plots showing the Pearson correlation coefficients for relationships between the concentrations of LDL or TCHO in peripheral blood and frequencies of PMN-MDSCs or All MDSCs. (B) Dot plots showing the Pearson correlation coefficients for relationships between the concentrations of LDL in peripheral blood and the frequencies of Ly6C+NK cells. (C) Dot plots (n=22) showing the Pearson correlation coefficients for relationships between the concentrations of LDL or TCHO i [file 12967_2021_3207_MOESM1_ESM.pdf]

Supplementary material

Supplementary Figures

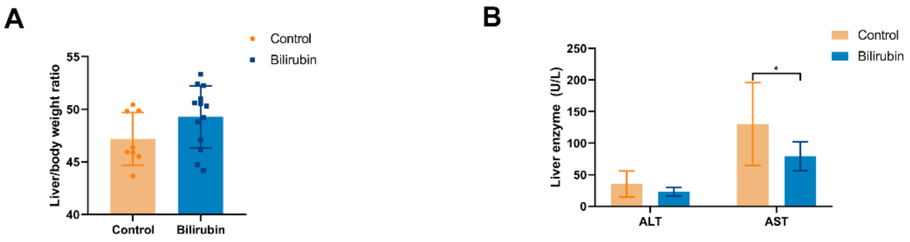

Figure S1. (A) ApoE$^{-/-}$ mice fed with a western-type diet were intraperitoneally injected with or without bilirubin. After ten weeks, mice were scarified, and the body and liver weight were measured. The relative liver weight was expressed as liver weight to body weight ratio. (B) Concentrations of liver enzymes were determined by biochemical analysis. Error bars represent mean ± SD. n (control) =8, n (bilirubin) =14. *$P < 0.05$. Differences were determined by an unpaired t-test.

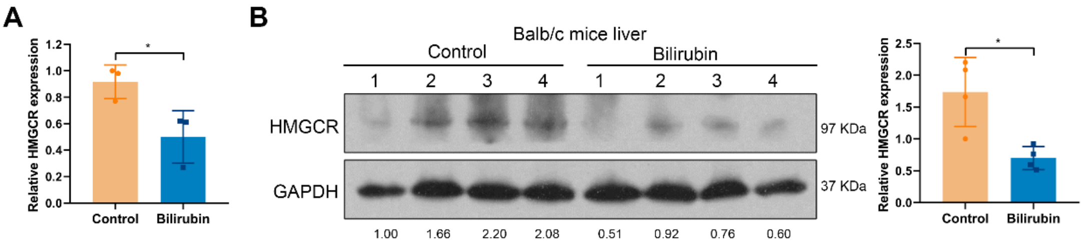

Figure S2. (A) Quantification of hepatic HMGCR protein expression in ApoE$^{-/-}$ mice treated with bilirubin (n=3) or vehicle (control, n=3). (B) BALB/c mice were received intraperitoneal injection of bilirubin (20 mg/Kg/day, n=4) or vehicle (n=4) once a day for 3 days and sacrificed at day 4. The expression levels of HMGCR in the livers were determined by western blot. Bar plots showing the relative HMGCR protein expression in the livers (right panel). *$P < 0.05$.

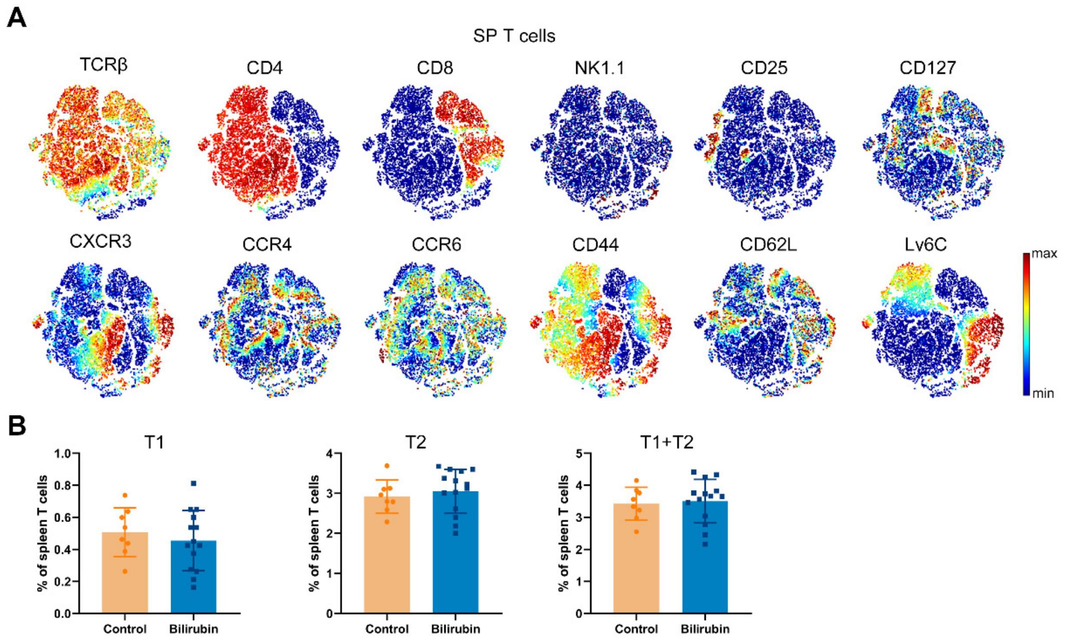

Figure S3. After determining the expression of 27 surface markers on the spleen cells using mass cytometry, the T cells expressing CD3 were gated and analyzed using viSNE. viSNE was used to visualize the distribution of the spleen T cells from both groups. Cells on the viSNE map were colored by normalized expression of indicated surface markers. (B) Bar plots showing the frequencies of T1 or T2 in spleen T cells obtained from ApoE$^{-/-}$ mice treated with or without bilirubin. Error bars represent mean ± SD. n (control) =8, n (bilirubin) =14.

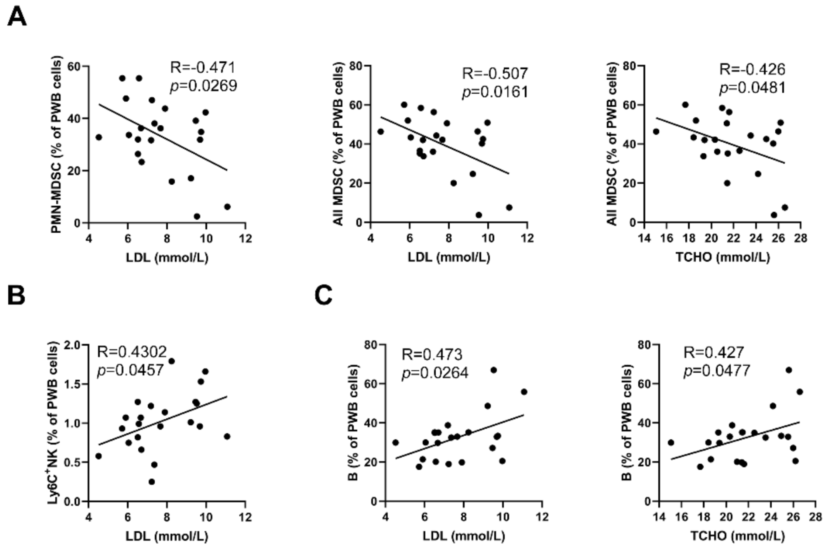

Figure S4. (A) Dot plots showing the Pearson correlation coefficients for relationships between the concentrations of LDL or TCHO in peripheral blood and frequencies of

PMN-MDSCs or All MDSCs. (B) Dot plots showing the Pearson correlation coefficients for relationships between the concentrations of LDL in peripheral blood and the frequencies of Ly6C^{+}NK cells. (C) Dot plots (n=22) showing the Pearson correlation coefficients for relationships between the concentrations of LDL or TCHO in peripheral blood and the frequencies of peripheral blood B cells. Correlations were determined by a Pearson test.

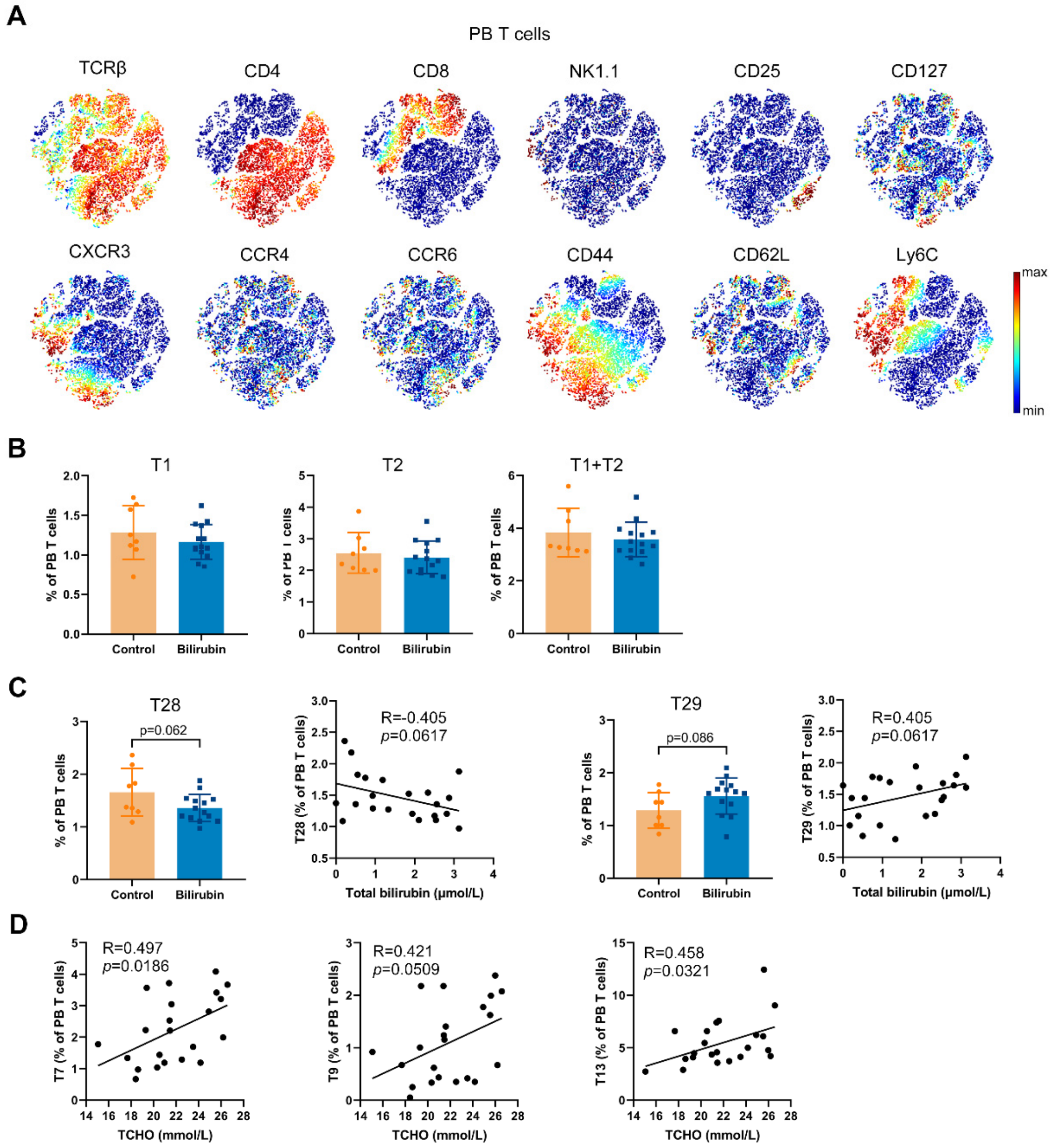

Figure S5. After determining the expression of 27 surface markers on the PWB cells using mass cytometry, the T cells expressing CD3 were gated and analyzed using viSNE. (A) viSNE was used to visualize the distribution of the peripheral blood T cells from both groups. Cells on the viSNE map were colored by normalized expression of

indicated surface markers. (B) Bar plots showing the frequencies of T1 or T2 in peripheral blood T cells obtained from ApoE-/- mice treated with or without bilirubin. (C) Bar plots showing the frequencies of T28 or T29 in peripheral blood T cells obtained from ApoE-/- mice treated with or without bilirubin (left panel). Dot plots showing the Pearson correlation coefficients for relationships between the concentrations of total bilirubin in peripheral blood and frequencies of T28 or T29 in peripheral blood T cells. (D) Dot plots (n=22) showing the Pearson correlation coefficients for relationships between the concentrations of TCHO in peripheral blood and the frequencies of T7, T9, T13 in peripheral blood T cells. Error bars represent mean ± SD. n (control) =8, n (bilirubin) =14. Differences were determined by an unpaired t-test. Correlations were determined by a Pearson test.

Supplementary Table

Table S1. Mass cytometry antibody reagents

**Label**	**Target**	**Ab clone**	**Dilution**	**Source**
89Y	CD45	30-F11	1:100	Fluidigm
141Pr	Ly6G	1A8	1:100	Fluidigm
142Nd	CD11c	N418	1:100	Fluidigm
143Nd	TCRβ	H57-597	1:100	Fluidigm
145Nd	CD4	RM4-5	1:100	Fluidigm
146Nd	F4/80	BM8	1:100	Fluidigm
148Nd	CD11b (Mac-1)	M1/70	1:100	Fluidigm
149Sm	CD19	6D5	1:100	Fluidigm
150Nd	Ly6C	HK1.4	1:100	Fluidigm
151Eu	CD25 (IL-2R)	3C7	1:100	Fluidigm
152Sm	CD3e	145-2C11	1:100	Fluidigm
156Gd	CD196 (CCR6)	29-2L17	1:100	Fluidigm
160Gd	CD62L (L-selectin)	MEL-14	1:100	Fluidigm
162Dy	CD44	IM7	1:100	Fluidigm
165Ho	CD161 (NK1.1)	PK136	1:100	Fluidigm
168Er	CD8a	53-6.7	1:100	Fluidigm
169Tm	CD206 (MMR)	C068C2	1:100	Fluidigm
170Er	CD49b	HMa2	1:100	Fluidigm
171Yb	CD80 (B7-1)	16-10A1	1:100	Fluidigm
172Yb	CD86	GL1	1:100	Fluidigm
174Yb	CD127 (IL-7Ra)	A7R34	1:100	Fluidigm
176Yb	CD45R (B220)	RA3-6B2	1:100	Fluidigm
163Dy	CD183(CXCR3)	CXCR3-173	1:100	BioLegend
175Lu	CCR4	2G12	1:100	BioLegend
159Tb	CD103	2E7	1:100	BioLegend
114Cd	IA/IE(MHC II)	M5/114.15.2	1:100	BioLegend
